# Spectral characteristics of urine specimens from healthy human volunteers analyzed using Raman chemometric urinalysis (Rametrix)

**DOI:** 10.1371/journal.pone.0222115

**Published:** 2019-09-27

**Authors:** Ryan S. Senger, Varun Kavuru, Meaghan Sullivan, Austin Gouldin, Stephanie Lundgren, Kristen Merrifield, Caitlin Steen, Emily Baker, Tommy Vu, Ben Agnor, Gabrielle Martinez, Hana Coogan, William Carswell, Lampros Karageorge, Devasmita Dev, Pang Du, Allan Sklar, Giuseppe Orlando, James Pirkle, John L. Robertson

**Affiliations:** 1 Department of Biological Systems Engineering, Virginia Tech, Blacksburg, Virginia, United States of America; 2 Department of Chemical Engineering, Virginia Tech, Blacksburg, Virginia, United States of America; 3 DialySenors, Inc., Blacksburg, Virginia, United States of America; 4 Veteran Affairs Medical Center, Salem, Virginia, United States of America; 5 Department of Statistics, Virginia Tech, Blacksburg, Virginia, United States of America; 6 Lewis-Gale Medical Center, Salem, Virginia, United States of America; 7 Department of Surgical Sciences – Transplant, Wake Forest University Baptist Medical Center, Winston-Salem, North Carolina, United States of America; 8 Department of Internal Medicine – Nephrology, Wake Forest University Baptist Medical Center, Winston-Salem, North Carolina, United States of America; 9 Department of Biomedical Engineering and Mechanics, Virginia Tech, Blacksburg, Virginia, United States of America; 10 Virginia Tech-Carilion School of Medicine and Research Institute, Blacksburg, Virginia, United States of America; Center for Molecular Biotechnology, ITALY

## Abstract

Raman chemometric urinalysis (Rametrix^™^) was used to analyze 235 urine specimens from healthy individuals. The purpose of this study was to establish the “range of normal” for Raman spectra of urine specimens from healthy individuals. Ultimately, spectra falling outside of this range will be correlated with kidney and urinary tract disease. Rametrix^™^ analysis includes direct comparisons of Raman spectra but also principal component analysis (PCA), discriminant analysis of principal components (DAPC) models, multivariate statistics, and it is available through GitHub as the Rametrix^™^ LITE Toolbox for MATLAB^®^. Results showed consistently overlapping Raman spectra of urine specimens with significantly larger variances in Raman shifts, found by PCA, corresponding to urea, creatinine, and glucose concentrations. A 2-way ANOVA test found that age of the urine specimen donor was statistically significant (p < 0.001) and donor sex (female or male identification) was less so (p = 0.0526). With DAPC models and blind leave-one-out build/test routines using the Rametrix^™^ PRO Toolbox (also available through GitHub), an accuracy of 71% (sensitivity = 72%; specificity = 70%) was obtained when predicting whether a urine specimen from a healthy unknown individual was from a female or male donor. Finally, from female and male donors (n = 4) who contributed first morning void urine specimens each day for 30 days, the co-occurrence of menstruation was found statistically insignificant to Rametrix^™^ results (p = 0.695). In addition, Rametrix^™^ PRO was able to link urine specimens with the individual donor with an average of 78% accuracy. Taken together, this study established the range of Raman spectra that could be expected when obtaining urine specimens from healthy individuals and analyzed by Rametrix^™^ and provides the methodology for linking results with donor characteristics.

## Introduction

Metabolomic analysis of normal human urine has identified over 2,000 separate chemical entities [1]. This and other -omics technologies, along with deep sequencing, have been used over the past decade in search of disease biomarkers, particularly for kidney disease [1–3]. In particular, biomarkers have been sought for chronic kidney disease (CKD) and end-stage kidney disease (ESKD), multifactorial diseases affecting up to 10% of the US population, and acute kidney injury (AKI), a significant cause of morbidity/mortality in hospitalized patients [4,5]. While a few candidate biomarker molecules for these diseases have been identified (cystatin C, periostin), these are not commonly measured (or readily available) to caregivers in patient care settings. This is due to expense, requirement for advanced technology (such as mass spectrometry), and lack of validation for the broad spectrum of clinical presentations of CKD and AKI [4–7].

In an alternative approach, we have invented and extensively validated a Raman spectroscopy-based technology called Raman chemometric urinalysis (Rametrix^™^) to analyze urine [8–10]. Raman spectroscopy is a mature, well-studied, and powerful technology [11] that has been applied to analysis of the chemical composition of a wide variety of solids and liquids, including biological specimens [8,9,12–15]. Rametrix^™^ captures Raman spectral signatures from hundreds of molecules in the urine simultaneously. It then compares these to those of other urine specimens and standards and identifies statistically significant differences. A proof-of-concept study demonstrated the capability of Rametrix^™^ to distinguish between urine specimens from healthy individuals and those from ESKD (CKD 4–5) patients receiving peritoneal dialysis therapy [9]. Thus, the potential for Rametrix^™^ includes the ability to screen for early signs of disease through a quick (i.e., less than 15 minutes) and inexpensive (i.e., few dollars per sample) Raman scan of a urine specimen. Current Raman instrumentation required is portable, has a small footprint (i.e., about the size of a laptop computer), and is relatively inexpensive (i.e., less than $20K). The purpose of this study was to use Rametrix^™^ to evaluate urine from healthy volunteers, both cross sectionally and longitudinally to establish a baseline or “range of normal” for when Rametrix^™^ is used to screen for the presence of disease. Our hypothesis was that Rametrix^™^ would be able to identify differences in urine samples from subjects based on age, sex, presence of menstruation, and over a 30-day collection cycle. Specific goals of this study included determining (i) what Raman shifts contribute to dataset variance; (ii) if observed variances can be correlated with age, sex, or a particular individual; (iii) what variations occur over a 30-day urine collection cycle for multiple healthy individuals; and (iv) if menstruation causes significant changes in the Raman spectra of urine.

## Materials and methods

### Approval and informed consent

This study was approved under Research Protocol VT15-703, administered by the Virginia Tech Institutional Review Board. Informed written consent was obtained for the collection of urine specimens from healthy volunteers. In accordance with this protocol, specimens were de-identified and assigned a code at the time of collection.

### Description of study population and sampling

Two hundred thirty-five (235) urine specimens were collected from 48 (39 females, 9 males) healthy human volunteers. For this study, “healthy” was defined as free of infectious, metabolic, or degenerative disease at the time of sample collection, and with no history/evidence of renal disease (based on laboratory serum creatinine measurements). Urine obtained from a healthy individual was referred to as “normal” urine, and the “range of normal” encompasses the variations in Raman spectra of normal urine. The age range of the population was 18 to 70 years, with 87.5% of the specimens from volunteers aged 19–22 years; the sample population median age was 21 years. Specimens were collected between January 8, 2017 and July 21, 2018. The sample size (235 urine specimens) was determined by the maximum number of healthy volunteers and specimens collected curing the collection period.

A thirty-day (30-day) urine specimen collection from 3 female and 1 male healthy donor volunteers is also included as a subset. First morning voids were collected each day, and repetitive collections were done to determine amount of variance (due to diet, lifestyle, and hydration, primarily) in urine molecular composition from the same individual over 30 days. These collection subsets were also used to determine if normal menstruation had a significant effect on the Raman molecular signature of normal urine, since the presence of blood in urine (hematuria) can be a sign of genitourinary pathology when not associated with menstruation.

### Specimen collection and storage

Free-catch (voided) urine specimens were collected in sterile 30 mL urine specimen cups, generally at the time of first daily urination following sleep. Following collection, specimens were refrigerated immediately and then stored at -35°C until analyzed. We determined the suitability of collection and storage conditions in a separate study of urine stability [5]. Unused portions of the specimen were stored at -35°C for the duration of the study and re-analyzed, as needed.

### Analytical standard

A synthetic urine analytical standard, Surine^™^ Urine Negative Control (Dyna-Tek Industries, Lenexa, KS) was obtained and used as a control reagent for sample measurements.

### Raman methodology and measurements

An Agiltron (Woburn, MA) PeakSeeker^™^ dispersive Raman spectrometer was used for analyses. The system was equipped to analyze bulk liquid samples, and a 785 nm (30 mW) laser excitation for 30 s with spectral resolution of 8 cm^-1^ was used.

Urine samples were equilibrated to 25°C, transferred to 1.5 mL glass vials, and placed in the spectrometer. Intensity data was collected over the Raman shift range of 250–1950 cm^-1^, which contains the accepted biological range where distinct signatures of biological molecules appear [16]. We have published similar methodology using a Bruker Senterra^™^ Raman microscope [12–15], and preliminary data analysis revealed the PeakSeeker^™^ analysis of liquid urine produced a detailed spectrum with simplified sample handling and analysis requirements. A minimum of 10 individual spectra were acquired per sample prior to data analysis.

### Computational methodology

Spectral baselining was done using the Goldindec algorithm [17], all scan replicates were averaged, and resulting spectra were vector normalized. Rametrix^™^ computations were applied using the Rametrix^™^ LITE Toolbox for MATLAB^®^ [9] and the Rametrix^™^ PRO Toolbox for MATLAB^®^ [10]. MATLAB^®^ r2018a (The MathWorks, Inc.; Natick, MA) with the Statistics and Machine Learning Toolbox was used for all Rametrix^™^ and statistical calculations.

### Public access

The Rametrix^™^ LITE Toolbox is offered under license agreement through GitHub (https://github.com/SengerLab/RametrixLITEToolbox). The Rametrix^™^ PRO Toolbox is also available through GitHub under license agreement (https://github.com/SengerLab/RametrixPROToolbox). Raman spectral data generated for this study are also shared through GitHub (https://github.com/SengerLab/Raman-Scans/tree/Normal-Urine).

## Results

### Raman spectra of urine

**Fig 1:**
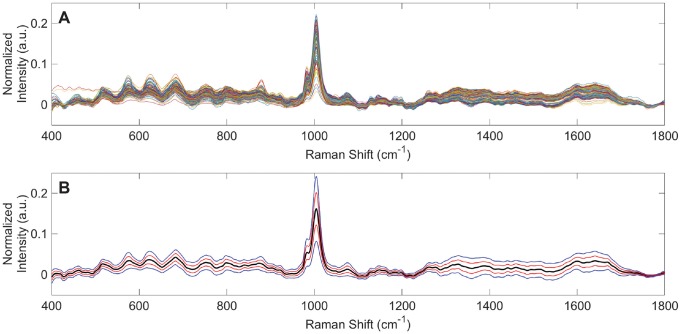
Raman spectra from 235 urine specimens from healthy individuals. (A) Overlaid vector normalized spectra. (B) The average urine spectrum (black) with ranges of 1 (red) and 2 (blue) standard deviations.

Raman spectra were obtained from 235 urine specimens of healthy individuals. Overlaid spectra, following baselining, replicate averaging, and vector normalization are shown in Fig 1A. An overall averaged Raman spectrum of normal urine was derived from these spectra and is shown in Fig 1B. This includes ranges of plus/minus one and two standard deviations from the average healthy urine spectrum. Evident in Fig 1 is notable variance in the Raman spectra at specific Raman shifts and ranges. These represent the range of normal for Raman spectra of urine from healthy individuals, and they were investigated further by principal component analysis (PCA) using the Rametrix^™^ LITE Toolbox for MATLAB^®^.

### Principal component analysis

**Fig 2:**
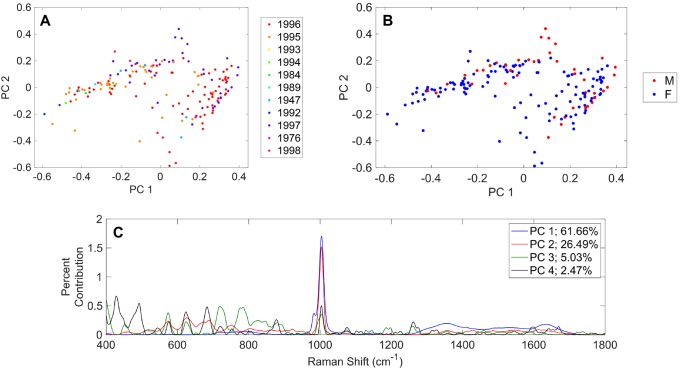
Principal component analysis (PCA) results for Raman spectra of 235 urine specimens from healthy individuals. PCA results based on (A) year individual was born and (B) sex of individual. (C) Contributions of Raman shift leading to separations among principal components.

PCA results are shown in Fig 2 and are coded by (A) birth year of the healthy individual and (B) sex (F = female; M = male). Initial inspection revealed no clustering according to birth year or sex, and these were investigated further through statistical analyses and model predictions (discussed later). The Rametrix^™^ LITE Toolbox for MATLAB^®^ enables identification of Raman shifts leading to the separation of data points in PCA. These are shown for the first four principal components in Fig 2C. Together, these first four principal components represent 95.7% of the dataset variance. The Raman shift at 1,002 cm^-1^ was the largest contributor to the dataset variance and was represented in multiple principal components (Fig 2C). In urine, this Raman shift is dominated by urea, suggesting the concentration of urea varies widely for healthy individuals. Other notable molecules identified in this figure are uric acid (981 cm^-1^), creatinine (680 cm^-1^), collagen (870 cm^-1^), and glucose (1,071 cm^-1^; 1,117 cm^-1^; and others) [16]. While these examples have been validated in our lab by scanning pure standards, research is ongoing to correlate more Raman shifts in Fig 2C with known urine metabolites [1].

### Discriminant analysis of principal components models

**Fig 3:**
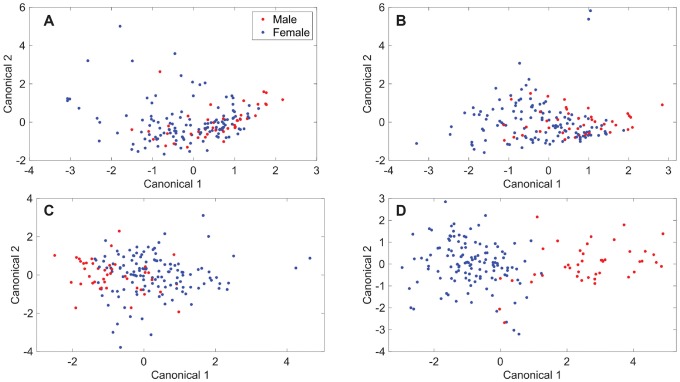
Discriminant analysis of principal components (DAPC) results. DAPC results for models made with the following number of principal components and percentage of dataset variability explained by those principal components: (A) 3, 90%; (B) 4, 95%; (C) 10, 99%; (D) 35, 99.9%.

The Rametrix^™^ LITE Toolbox for MATLAB^®^ allows the construction of discriminant analysis of principal components (DAPC) models that can be used to further cluster Raman spectra based on specified attributes. This was applied to the dataset of Raman spectra from healthy individuals to determine whether true differences exist between urine specimens based on sex. The DAPC models were constructed using different numbers of principal components and are shown in Fig 3. Increasing the numbers of principal components allowed more of the dataset variance to be included in the DAPC models and led to better clustering and separation between the spectra based on sex. Similar to PCA results, in DAPC models (Fig 3), each point represents an entire Raman spectrum, and clustering occurs among spectra with similarities. The DAPC models were constructed to represent 90% of the dataset variance with three principal components (Fig 3A); 95% of dataset variance with four principal components (Fig 3B); 99% with ten principal components (Fig 3C); and 99.9% with thirty-five (35) principal components (Fig 3D). Visual separation of clusters based on sex was apparent as at least 99% of the dataset variance was included in the DAPC models. Next, it was tested whether these models could predict whether an unknown urine specimen was from a female or male donor.

The Rametrix^™^ PRO Toolbox for MATLAB^®^ was developed to make predictions of unknown specimens using DAPC models built in the Rametrix^™^ LITE Toolbox and to evaluate these models using a leave-one-out build/test routine. Here, DAPC models were built using all but one of the data points (i.e., spectra) in the dataset. The classification (i.e., donor sex) of that remaining spectrum was predicted using the DAPC models, and it was recorded whether or not the prediction was correct. This routine was then repeated for every spectrum in the dataset. From there, the accuracy, sensitivity, and specificity of the model predictions were calculated. The model accuracy refers to the percentage of the total number of data points that were predicted correctly. The sensitivity refers to the true positive rate. In this case, it was the percentage of specimens from females that were identified correctly as female. The specificity refers to the true negative rate. In this case, it was the percentage of specimens from males that were identified correctly as male. Results are shown in Table 1. Accuracy ranges from 71–84% were obtained for the four DAPC models tested. However, the DAPC model consisting of 10 principal components (representing 99% of the dataset variance) performed best in terms of accuracy (71%), sensitivity (72%), and specificity (70%). While other models had better accuracy and sensitivity, their specificity values were well below 70%. The DAPC model consisting of 35 principal components (representing 99.9% of the dataset variance) showed signs of overfitting the data (i.e., improved DAPC clustering but reduced accuracy and/or specificity in leave-one-out trials). Together, this means that Rametrix^™^ can predict whether a urine specimen from a healthy individual belonged to a female or male with about 70% accuracy. This is far better than the 50% probability assigned by chance.

**Table 1:** Rametrix^™^ PRO results showing the ability to predict whether an unknown urine specimen came from a female donor.

Percent Variability Explained by Principal Components	Number of Principal Components used in DAPC Model	Accuracy*	Sensitivity*	Specificity*
90%	3	77%	94%	26%
95%	4	71%	76%	56%
99%	10	71%	72%	70%
99.9%	35	84%	100%	37%

*Predictions were from a leave-one-out training/testing routine.

### Statistical analysis

The entire spectral dataset was analyzed by 2-way ANOVA to determine if the factors “sex” (i.e., female or male) or “birth year” of the specimen donor were statistically significant. To do this, Raman spectra had to be reduced to a single number per spectrum. Previously, we explored three ways of doing this, and determined that calculation of the Total Spectral Distance (TSD) and Total Principal Component Distance (TPD) were most adequate [8]. The following is a brief explanation of the TPD calculation. PCA reduces a complex Raman spectrum, composed of intensity value for each Raman shift between (400–1800 cm^-1^), to a single number computed from principal components. The principal components are ranked by the amount of dataset variance that they can explain, meaning that Raman spectra encompassing many intensity data points can be represented by only a few principal components. For urine specimens in this dataset, four principal components were found to contain more than 95% of the dataset variance (see Fig 2C). With a urine specimen reduced to its four principal components, it can be compared with the urine analytical standard Surine^™^ using its four principal components and the distance formula, as shown in Eq 1, where *P*_*u*,*i*_ is the i^th^ principal component of a urine specimen, and *P*_*control*,*i*_ is the i^th^ principal component of Surine^™^ (i.e., the control).

$$TPD=\sum_{i=1}^{4}\sqrt{{\left(P_{u,i}-P_{control,i}\right)}^{2}}$$ (1)

TPD data values for this study have been made available through GitHub (see Public Access section). 2-Way ANOVA results for TPD given factors of sex and birth year are shown in Table 2. Initially, the interaction of sex and birth year was considered in the 2-way ANOVA, and this was found to be insignificant (p = 0.166). Then, 2-way ANOVA was repeated without the interaction term included, and this returned p-values of 0.0526 for sex and <0.001 for birth year. This suggest that significant differences in Raman spectra of urine from healthy individuals change significantly with age and possibly significantly with sex, depending on the confidence level chosen. This correlates with the accuracy, sensitivity, and specificity obtained for predicting whether the urine specimen donor was female or male (Table 1).

**Table 2:** 2-Way ANOVA results based on TPD calculations.

Source	Sum of Squares	Degrees of Freedom	Singular?	Mean Squares	F-Statistic	p-Value
Sex (F/M)	0.0678	1	1	0.0677	3.81	0.0526
Birth Year	0.770	10	1	0.0770	4.33	2.25E-05
Error	2.86	161	0	0.0178	n/a	n/a
Total	3.71	173	0	n/a	n/a	n/a

### 30-day study results

Three healthy females and one male contributed first morning void urine specimens every day for 30 days. The specimens collected during days of menstruation were identified by the female donors. With these data the following questions were addressed:

1. What is the TPD range for each healthy individual over the 30-day collection period?
2. Are the Raman spectra statistically different based on donor?
3. Can Rametrix^™^ PRO be used to determine whether a urine specimen was collected during menstruation?
4. Can Rametrix^™^ PRO be used to link an unknown urine specimen to the donor?

The TPD was calculated for each urine specimen, and the average, range, and standard deviation values per donor are given in Table 3. A 2-way ANOVA test was applied to the 30-day dataset and revealed that the individual donor was statistically significant (p < 0.001), but the presence of menstruation was not (p = 0.695). The factor interaction term did not apply (p = 0.294). Next, pairwise comparisons were applied using Tukey’s honestly significant difference test. These revealed that specimens from donor Female 3 were statistically different (all p-values ≤ 0.0022) from those of all other donors. Specimens from all other donors (other than Female 3) were found not statistically different from one another (all p-values > 0.059).

**Table 3:** TPD results for the 30-day study.

30-Day Donor	Average TPD* (± 1 s.d.)	TPD Range
Female 1	0.301 ± 0.111	0.0391 − 0.516
Female 2	0.225 ± 0.151	0.00831 − 0.421
Female 3	0.107 ± 0.101	0.00843 − 0.385
Male 1	0.257 ± 0.140	0.0151 − 0.492

*Average TPD values are given ± 1 standard deviation (s.d.).

Next, Rametrix^™^ PRO was tested to determine if it can identify a urine specimen collected during menstruation and if it can correlate an unknown urine specimen with the donor. Results are given in Table 4 for DAPC models constructed using 9 PCs (representing 99% of the dataset variability) and 30 PCs (representing 99.9% of the dataset variability). Similar to the Rametrix^™^ PRO results shown in Table 1, using 99% of the dataset variability provided better results, even though the visual clustering was more distinct when using 99.9% of the dataset variability. Results indicate that specimen collection during menstruation cannot be identified in this dataset using Rametrix^™^ PRO (10% sensitivity in Table 4). This also indicates that menstruation was not responsible for skewing Rametrix^™^ urine screening results dramatically in this dataset, which is consistent with ANOVA test results. For the four healthy individual donors (3 female, 1 male) in the 30-day study, Rametrix^™^ PRO was able to correctly correlate an unknown urine specimen with the donor with an average of 78% accuracy (62% sensitivity; 84% specificity). This is well above the random chance probability of 25%. Finally, for each individual donor in the 30-day study, no distinct pattern was observed for how urine specimens changed over the course of the 30 days.

**Table 4:** Rametrix^™^ PRO results for the 30-day study.

	Accuracy*	Sensitivity*	Specificity*
*99% Variability Explained by Principal Components (9 PCs)*			
Menstruation	91%	10%	98%
Female 1	75%	57%	81%
Female 2	80%	58%	89%
Female 3	84%	57%	92%
Male 1	74%	77%	73%
*99*.*9% Variability Explained by Principal Components (30 PCs)*			
Menstruation	11%	100%	3%
Female 1	77%	7%	100%
Female 2	74%	3%	100%
Female 3	81%	23%	100%

*Predictions were from a leave-one-out training/testing routine.

## Discussion

Determining what is normal is critical for identifying what is abnormal. Ultimately, Rametrix^™^ analysis of urine will be used to screen for the presence of diseases, and our previously-published proof-of-concept with healthy individuals and ESKD patients [9] supports this notion. However, the variance in Raman spectra of urine specimens from healthy individuals needs to be known for more reliable datasets of Raman spectra of normal urine to be constructed and validated. This study represents a first attempt to obtain this range of normal for use with Rametrix^™^, a Raman spectroscopy-based technology. Interestingly, in this expanded study (relative to our proof-of-concept) with 235 urine specimens from healthy individuals, different levels of spectral variance were observed at different Raman shifts. PCA with the Rametrix^™^ LITE Toolbox for MATLAB^®^ helped determine which Raman shifts were most significant in accounting for differences between urine specimens. Many of these correlated with well-characterized urine components, such as urea, creatinine, and glucose, but there are several other bands yet to be identified (Fig 2C). The use of previously published urine metabolomics and Raman spectral libraries are being used to identify these remaining metabolites, but adequate standards and validations are still needed.

With this dataset of Raman spectra from healthy individuals, Rametrix^™^ analysis was able to answer other questions, such as:

1. Does the age of the urine specimen donor impact urine molecular composition and the resulting Raman spectrum?
2. Does the identified sex (female or male) play a role?
3. Are significant differences observed should a urine specimen be given during menstruation?
4. What level of variance should be expected from a 30-day collection cycle from the same individual?

Specifically, we found that significant differences (p < 0.001) in Raman spectra of a urine specimen can be attributed to the age of the donor, even if the donor is in good health. Fewer differences (p = 0.0526) were observed based on the sex of the donor, and the ability to predict the sex of the donor of an unknown urine specimen was attempted. The accuracy (71%) of predicting donor sex using Rametrix^™^ PRO, sensitivity (72%), and specificity (70%) were better than random chance (50%). While this result may not yet be relevant clinically, it demonstrates the degree to which differences according to sex are real and discernable. ANOVA results revealed the significance of these factors, and the Rametrix^™^ PRO results demonstrate the degree of overlap that exists. In addition, donor characteristics, such as age and sex, may play important roles in future Rametrix^™^ screens that identify the presence of disease. Additionally, the 30-day study subset of urine specimens revealed menstruation did not contribute statistically significant changes to the Rametrix^™^ spectral signature of urine (p = 0.695). Also, individuals collected over 30 days showed variations, but we were unable to establish correlations to diet and lifestyle at this point. Knowing the range of normal variation is also critical should Rametrix^™^ be used to screen urine specimens for the presence of disease and/or track patient progress in response to treatment. Clearly, more work is needed in relating these variations to diet and lifestyle. Furthermore, repeated collections from an individual may be used to define what is normal for that individual. Rametrix^™^ PRO was able to correlate an unknown specimen with the donor in the 30-day study with 78% accuracy (62% sensitivity; 84% specificity). This further supports that establishing a range of normal for an individual may hold value when using Rametrix^™^ to screen for the presence of disease, as each individual may have a slightly different version of normal.

From here, this dataset of Raman spectra of urine specimens from healthy individuals will be used in expanded studies to compare against those obtained from patients with kidney and urinary tract diseases. This approach will ultimately lead to Rametrix^™^ being used to screen for the presence of diseases and track the progress of treatments.
